# Supplementary material for: Prevalence of Culturable Bacteria and Yeasts in the Nasopharynx Microbiota during the Physiological Course of Pregnancy
Source: J Clin Med. 2023 Jul 2;12(13):4447. doi: 10.3390/jcm12134447 (PMC10342925; doi:10.3390/jcm12134447)
Supplement: Supplementary file 1 [file jcm-12-04447-s001.zip › jcm-2420055-supplementary.pdf]

Supplementary

Table S1. The level of bacterial and/or fungal species colonized the nasoparyngeal cavity of each one case in the PW group compared to NPW group on basis of results of the nonparametric Mann-Whitney U test

	Descriptives				
**Colonization with**	***U***	***p***	***Min***	***Max***	***Me***
Gram-positive bacteria	752.00	0.675			
PW			0.00	6.00	2.00
NPW			0.00	5.00	2.00
Gram-negative	728.50	0.422			
PW			0.00	2.00	0.00
NPW			0.00	2.00	0.00
Gram-negative oxidase-positive	760.50	0.472			
PW			0.00	1.00	0.00
NPW			0.00	1.00	0.00
Gram-negative oxidase-negative	741.00	0.421			
PW			0.00	1.00	0.00
NPW			0.00	1.00	0.00
all bacteria	786.00	0.933			
PW			1.00	6.00	2.00
NPW			1.00	6.00	2.00
yeasts	765.00	0.292			
PW			0.00	1.00	0.00
NPW			0.00	0.00	0.00
all isolates	780.50	0.890			
PW			1.00	6.00	2.00
NPW			1.00	6.00	2.00
Staphylococcus spp.	753.50	0.670			
PW			0.00	5.00	1.00
NPW			0.00	3.00	1.00
Streptococcus spp.	721.00	0.121			
PW			0.00	2.00	0.00
NPW			0.00	1.00	0.00
other Gram-positive	754.00	0.673			
PW			0.00	4.00	0.00
NPW			0.00	2.00	0.00
Enterobacteriaceae	722.50	0.205			
PW			0.00	1.00	0.00
NPW			0.00	1.00	0.00
Erwiniaceae	791.50	0.930			
PW			0.00	1.00	0.00
NPW			0.00	1.00	0.00
Pseudomonadales	787.00	0.863			
PW			0.00	1.00	0.00
NPW			0.00	1.00	0.00

	Descriptives				
**Colonization with**	***U***	***p***	***Min***	***Max***	***Me***
other Gram-negative	773.00	0.672			
PW			0.00	1.00	0.00
NPW			0.00	1.00	0.00

U, test statistics; *p,* statistical significance; *Me,* median; *Min,* minimum value; *Max,* maximum value; PW, Pregnant women group; NPW, Non-pregnant women group. Gram-variable cocci and the bacterial families *Morganellaceae* and *Raoultella* due to insufficient numbers (one case each) were excluded from the study

Table S2. The effect of selected predictors on *Enterobacteriaceae* family colonization in nasopharynx cavity.

**Bacteria**	**Regression coefficients**					
	**Predictor**	***B***	***Z***	***P***		***OR***
Enterobacteriaceae	Intercept	4.93	1.71	0.087		138.51
	**Age**	**-0.23**	**-2.33**	**0.020**	*	**0.79**
	health condition	-0.84	-1.02	0.310		0.43
	place of residence	0.06	0.08	0.938		1.06
	exposure to smoke	-0.03	-0.03	0.976		0.98

B – estimate; *Z* – test statistics; *p* – statistical significance; *OR* – odds ratio;* $p < 0.05$

Table S3. The effect of age on the occurrence of *Enterobacteriaceae* Gram-negative rods.

				**95% Confidence Interval**	
**Age**		***P***	***SE***	**Lower limit**	**Upper limit**
23.91	⁻	0.21	0.07	0.11	0.38
30.33	µ	0.06	0.03	0.02	0.17
36.75	⁺	0.01	0.02	0.00	0.12

P – probability; *SE* – standard error; $^{-}$ mean - 1*SD*, $^{\mu}$ mean, $^{+}$ mean + 1*SD*

Table S4. Bacteria and yeasts prevalence in the nasopharynx of throughout the total study sample (n=83) with selected factors as potent predictors, including age, health status, place of residence and exposure to tobacco smoke.

Dependent variable	Model			
	R^2	χ^2	*df*	*P*
**Gram-positive bacteria**	0.10	4.50	4	0.342
Staphylococcus spp.	0.03	1.64	4	0.801
Streptococcus spp.	0.14	6.18	4	0.186
Cutibacterium spp.	0.41	13.03	4	**0.011***
other Gram-positive	0.02	1.77	4	0.778
**Gram-negative bacteria**	0.03	2.67	4	0.614
Gram-negative oxidase-positive	0.20	7.37	4	0.117
order: *Pseudomonadales*	0.20	7.37	4	0.117
Gram-negative oxidase-negative	0.09	6.49	4	0.166
family: *Enterobacteriaceae*	0.17	9.63	4	**0.047***
family: *Erwiniaceae*	0.15	3.99	4	0.408
other Gram-negative	0.02	0.88	4	0.928
**Yeasts**	0.23	4.26	4	0.372

The explanatory variables in each of the models were age, health status, place of residence and exposure to tobacco smoke (current active smoking or exposure to secondhand smoke indicates exposure to tobacco smoke). Logistic regression models were used; R^2 – model fit factor; χ^2 – test statistics; *df* – degrees of freedom; *p* – statistical significance (* $p < 0.05$).
